# Superior Vena Cava Syndrome Due to Thrombosis: A Case Report

**DOI:** 10.7759/cureus.24811

**Published:** 2022-05-07

**Authors:** Muhammad S Haider, Madiha F Master, Sobha Atluri, Jay Nfonoyim

**Affiliations:** 1 Internal Medicine, Richmond University Medical Center, New York, USA; 2 Medical School, Philadelphia College of Osteopathic Medicine, Philadelphia, USA; 3 Pulmonary and Critical Care, Richmond University Medical Center, Staten Island, USA

**Keywords:** rare clinical entity, anti-coagulation, anaplastic thyroid cancer, thrombosis, superior vena cava (svc) syndrome

## Abstract

Superior vena cava (SVC) syndrome is a clinical entity with signs and symptoms resulting from obstruction of blood flow through the SVC. The resulting obstruction leads to edema in the upper body, including the head, neck, and upper extremities. Clinical signs and symptoms can include plethora, cyanosis, dyspnea, stridor, cough, and hoarseness, as well as more serious complications such as cerebral edema leading to headache, confusion, and coma. Here, we present an interesting case of a 66-year-old female, with a medical history of esophageal cancer in remission and thyroid cancer currently undergoing radiation therapy, who was admitted for facial and upper extremity swelling. The initial impression was of angioedema or an allergic reaction. Imaging studies showed thrombus in the SVC resulting in SVC syndrome. The patient was treated with heparin initially, with a plan for an interventional radiologist to perform catheter-guided thrombolysis. However, the patient became unstable and ended up requiring mechanical ventilation. The patient was eventually discharged on oral anticoagulants. This case was rare as the patient developed SVC syndrome from venous thrombosis in the absence of any external tumor compression or as a result of an intravascular catheter.

## Introduction

Superior vena cava (SVC) represents the largest vein in the mediastinum, formed by the merging of the right and left brachiocephalic vein and drains blood from the upper part of the body. SVC syndrome results from obstruction of blood flow in the SVC, which can either be from external compression or an internal blockage from a thrombus or tumor infiltration. It has been associated with malignancies, such as lymphoma, small-cell lung cancer, and non-small-cell lung cancer, as well as metastatic lesions. SVC syndrome resulting from clots is usually linked to venous catheters and pacemaker leads. On rare occasions, patients in hypercoagulable state, that is, those with underlying malignancy, may develop spontaneous thrombosis of the vessel without having any venous catheters or leads. The most frequent clinical findings include swelling of the face, neck, and upper extremities. Other signs and symptoms include hoarseness, chest pain, dysphagia, changes in mental status, headache, syncope, and periorbital edema. Although patients can have gradual progression of symptoms, it can often be life-threatening in cases with acute onset. Treatment depends upon the underlying cause the and severity of the symptoms.

## Case presentation

A 66-year-old Caucasian female with a medical history of hypertension, diabetes mellitus, asthma, pulmonary embolism, and lower extremity deep venous thrombosis being treated with rivaroxaban; esophageal cancer in remission; and anaplastic thyroid cancer being treated with radiation therapy presented to the emergency department with chest pain, swelling of the lips and throat, dilated superficial facial veins, and bilateral upper extremity swelling.

On admission, her blood pressure was 120/75 mmHg, heart rate was 90 beats per minute, respiratory rate was 15 breaths per minute, temperature was 99.7°F, and saturation of 100% on room air. The initial chest X-ray showed no evidence of mass or interstitial lung disease. Lab investigation showed anemia with a hemoglobin of 8.5 g/dL, aspartate aminotransferase of 86 U/L, blood urea nitrogen of 24 mg/dL, and creatinine of 1.4 mg/dL. Prothrombin time, activated partial thromboplastin time, and international normalized ratio (INR) were within normal limits. Moreover, troponins and electrocardiogram showed no signs of cardiac ischemia.

**Figure 1:**
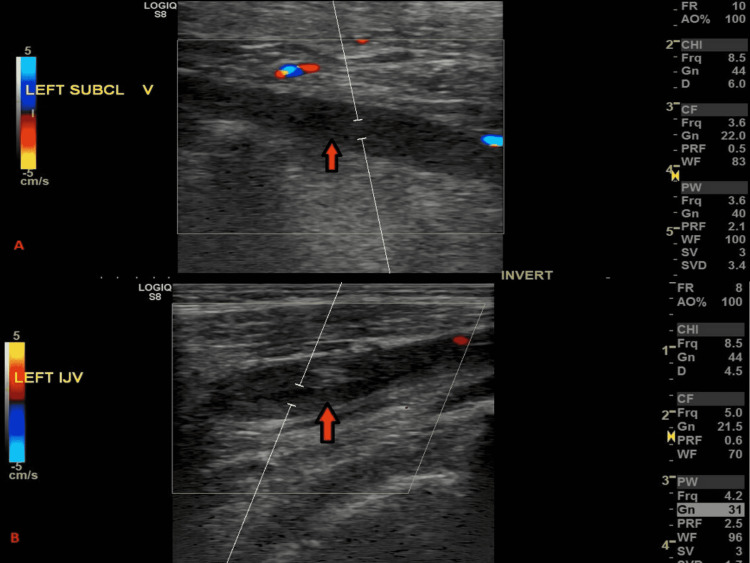
Venous duplex ultrasound showing clots (arrows) in the left subclavian vein (A) and the left jugular vein (B).

The patient was initially treated for angioedema with dexamethasone and diphenhydramine as findings were suspicious for angioedema secondary to the patient’s home medications of enalapril. Venous duplex ultrasound of the upper extremities was performed which showed acute clots in the left jugular vein and the subclavian vein, with no clots in the right upper extremity (Figure 1).

The patient was started on enoxaparin, and computed tomography (CT) of the chest/abdomen/pelvis was ordered to rule out SVC syndrome from local advancement of anaplastic thyroid cancer.

**Figure 2:**
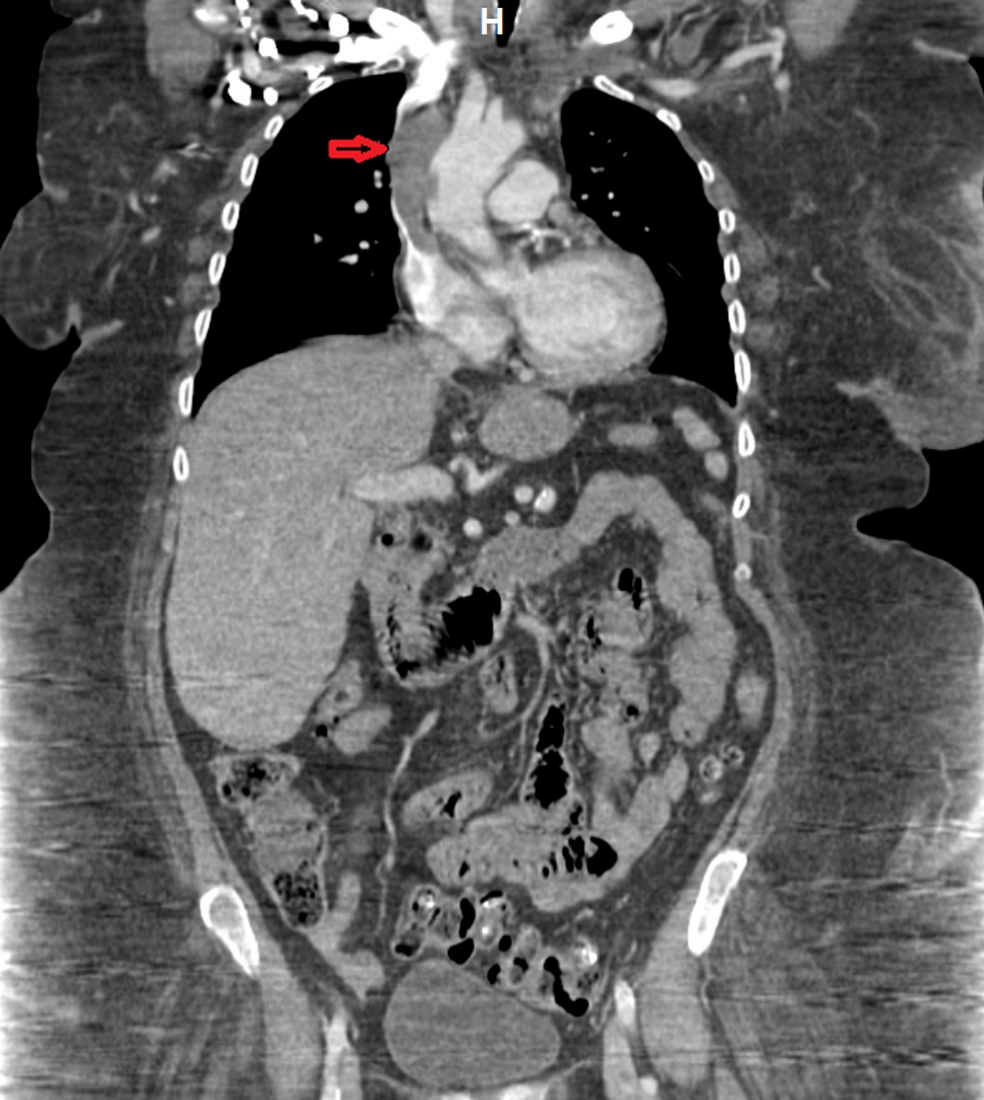
CT of the chest/abdomen/pelvis (coronal view). The red arrow shows soft tissue density in the SVC with contrast flowing around the periphery consistent with acute SVC thrombosis. CT: computed tomography; SVC: superior vena cava

The patient was pre-treated with hydrocortisone and diphenhydramine due to an iodine-based contrast allergy. She underwent CT of the chest/abdomen/pelvis with intravenous contrast which showed a soft tissue density in the SVC with contrast flowing around the periphery. The findings were consistent with acute SVC thrombosis. No secondary collateralization was noted, with thrombus also likely present in the left innominate vein (Figure 2).

The patient was started on intravenous heparin in place of enoxaparin. Although her symptoms remained unchanged, she started requiring increased oxygen support.

**Figure 3:**
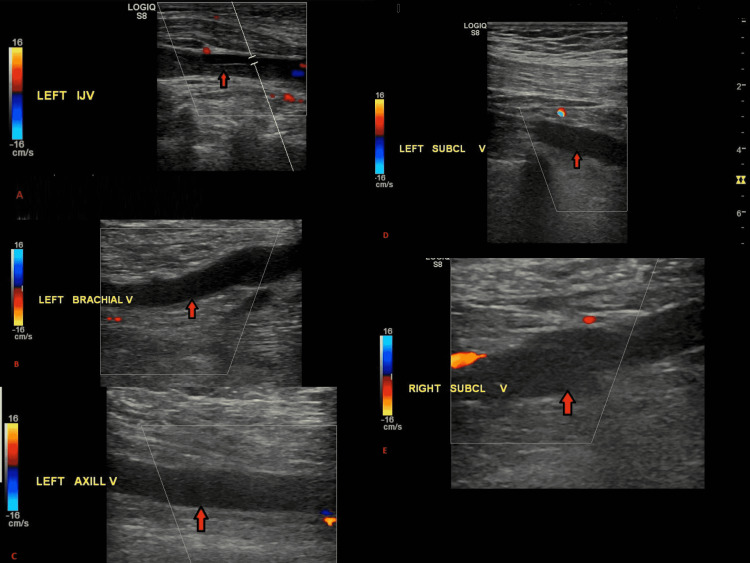
Venous duplex ultrasound showing clots in the left internal jugular (A), left subclavian (B), left axillary (C), and (D) left brachial veins. A clot can also be seen in the right subclavian vein (E).

Vascular surgery and interventional radiology were consulted who recommended catheter-guided tissue plasminogen activator. The patient’s left arm swelling started to worsen, with new-onset pain and discoloration. The patient continued to be in respiratory distress requiring supplemental oxygen. Her hemoglobin decreased to 6.7 g/dL, following which we transfused one unit of packed red blood cells. A repeat venous duplex ultrasound of the upper extremities showed worsening of clots in the left internal jugular vein and subclavian vein, with new clots in the axillary vein and the brachial veins, as well as in the right subclavian vein (Figure 3).

**Figure 4:**
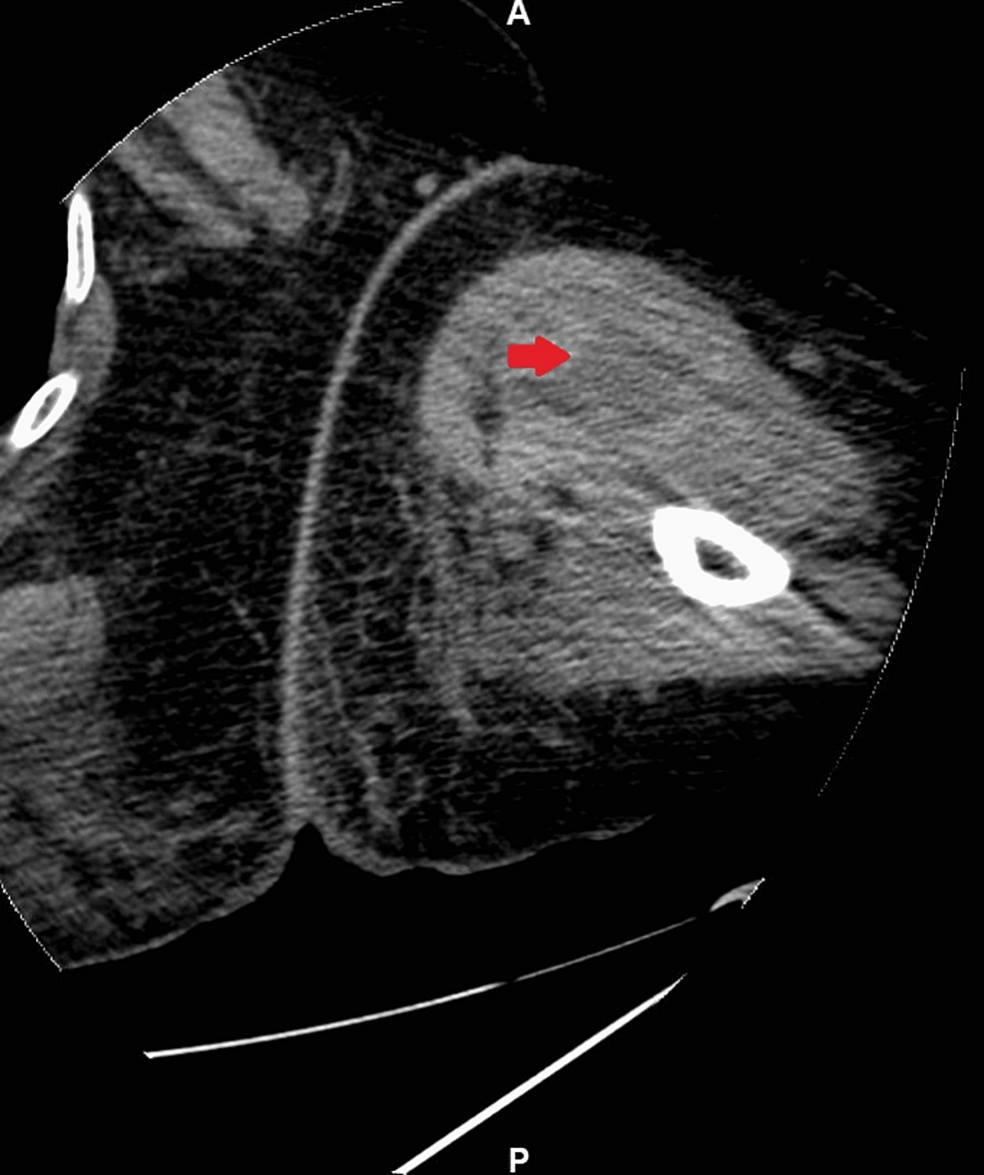
CT of the left upper extremity without contrast showing a 4 × 6 × 10 cm hypoattenuating mass in the biceps muscles showing possible hematoma.

A CT of the left upper extremity was also done which showed a 4 × 6 × 10 cm hypoattenuating mass in the biceps muscle most likely representing a hematoma (Figure 4).

**Figure 5:**
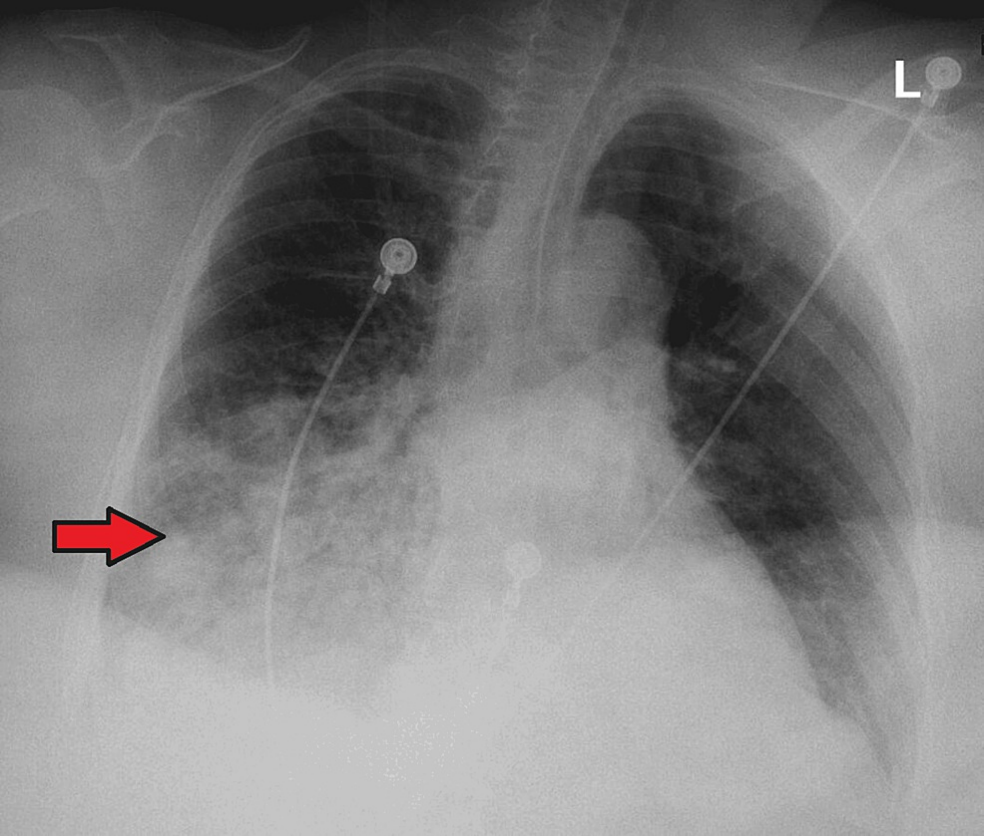
Anteroposterior view showing new extensive confluent opacity (arrow) involving the inferior one-half of the right hemithorax and is highly suggestive of aspiration. The left lung remains grossly clear.

The patient underwent CT angiography (CTA) of the upper extremity to locate the source of the bleed; however, immediately after the CTA, she likely had an allergic reaction to iodine contrast. She started wheezing, became hypoxic with oxygen saturation dropping to 80%, and was placed on bilevel positive airway pressure (BIPAP). The patient also felt nauseous and vomited. She was subsequently intubated due to acute hypoxic respiratory failure, low Glasgow Coma Scale score, and the inability to protect her airways. A post-intubation chest X-ray also showed signs of pneumonia likely from aspiration (Figure 5).

The patient had elevated D-dimers but normal fibrinogen levels. An echocardiogram showed an ejection fraction of 60% with grade 1 diastolic dysfunction and no right heart strain. Phosphatidylserine (immunoglobulin (Ig)G, IgA, IgM), cardiolipin antibodies (IgG, IgA, and IgM), complement C3, and complement C4 were low. Factor V Leiden mutation and lupus anticoagulant were not detected.

Heparin was kept on hold due to the increased risk of bleeding and to prevent further worsening of hematoma. The patient was eventually extubated, and once hemoglobin became stable, heparin was restarted. The patient continued to require supplemental oxygen to maintain adequate oxygen saturation. Her condition again deteriorated with worsening hypoxia and she went into pulseless electrical activity, which led to her being re-intubated. She was successfully extubated the second time after being on mechanical ventilation for four days. She was eventually weaned off supplemental oxygen. Given the high risk associated with the intravascular intervention, such as pulmonary embolism, the patient decided to pursue medical management only. She was eventually discharged from the hospital on apixaban and was asked to closely follow up with her hematologist-oncologist as outpatient.

## Discussion

SVC syndrome is a result of blood flow occlusion through the SVC due to external compression, internal obstruction, or vessel wall tumor infiltration. In 1757, the first case of SVC syndrome was described in a patient having a large syphilitic aneurysm [1]. In the United States, around 15,000 cases of SVC syndrome are reported every year [2].

In the past, >90% of SVC syndrome cases were associated with malignancy [3]. The most frequent cause is mediastinal malignancies, primarily small-cell bronchogenic carcinoma [4]. The second most common malignancy associated with SVC syndrome is non-Hodgkin’s lymphoma, followed by metastatic tumors, breast cancer, germ cell tumors, and sarcoma, among others [5] Recently, approximately 35% of SVC syndrome cases have been attributed to benign etiology which includes nonmalignant conditions or thrombosis associated with increased use of intravascular devices such as catheters and pacemakers [6].

The incidence of SVC syndrome due to the development of acute and spontaneous thrombus, in the absence of extrinsic tumor compression or intravascular device, is extremely rare. Patients with thyroid cancer, similar to our case, are at risk of thrombosis either due to compression [7,8] through angioinvasion [9,10] or because of a prothrombotic state. A literature review reported the association between paraneoplastic thrombosis in the SVC in patients diagnosed with other types of cancer including lung cancer, renal cell cancer, and ovarian carcinoma [11-13].

An extensive history and physical examination are essential to diagnose SVC syndrome. Symptoms associated with SVC syndrome can be divided into the following four categories: neurological, laryngopharyngeal, facial and chest wall, and upper extremities [14]. The most common clinical signs include face/neck swelling, upper extremities swelling, distended neck veins, cough, orthopnea, dyspnea, distended chest vein, and conjunctival suffusion [4]. Our patient presented with similar symptoms which, according to the patient, developed rapidly overnight. Ultrasound-guided Doppler of jugular, innominate, and subclavian veins is useful for identifying thrombus within the vessel lumen. For a definitive diagnosis of venous obstruction due to SVC syndrome, contrast venography and CT angiography are used [15].

Traditionally, SVC syndrome was treated with open surgical repair through bypass grafting with femoral vein, spiral saphenous vein, dacron graft, or polytetrafluoroethylene graft [16]. However, now it is mainly managed by treating the underlying etiology. Appropriate radiation and chemotherapy are considered for patients suffering from SVC syndrome caused by obstruction from a compressing tumor [17]. Stent placement is being increasingly used to relieve compression of the vessel by tumors [18]. For SVC syndrome caused due to thrombus relating to an indwelling intravascular device, primarily the intravascular device is removed along with the initiation of anticoagulation therapy and catheter-directed thrombolysis [19].

In a rare scenario of SVC thrombosis, the preferred treatment is anticoagulation. The majority of clinicians recommend thrombolytics before initiating anticoagulation. We also planned to perform intravascular thrombolysis in our patient but were not able to because of the hemodynamic instability of the patient. Rapid relief of symptoms can also be achieved with balloon angioplasty with stenting, making it a first-line therapy in acute thrombosis-related SVC obstruction [15].

## Conclusions

SVC syndrome results from conditions leading to obstruction of blood flow through the SVC. Our case highlights a rare presentation of SVC syndrome due to thrombus in the SVC. The case also highlights the fact that SVC syndrome should be considered in patients with a history of malignancy who present with upper extremity and facial swelling, and it should be a part of differential diagnosis for clinicians who encounter such patients. The case also helps to emphasize the challenge in managing these patients due to complications associated with this condition and how a multidisciplinary approach is needed to better manage these patients.
